# Title-blended supervision models for post-graduate rural generalist medical training in Australia: an interview study

**DOI:** 10.1186/s12909-022-03529-x

**Published:** 2022-06-20

**Authors:** Priya Martin, Belinda O’Sullivan, Carla Taylor, Glen Wallace

**Affiliations:** 1 General Practice Supervisors Australia, PO Box 141, Bendigo North, VIC 3550 Australia; 2 Rural Clinical School, Faculty of Medicine, University of Queensland, Locked Bag 9009, Toowoomba, QLD 4350 Australia; 3 School of Rural Health, Monash University, PO Box 666, Bendigo, VIC 3550 Australia; 4 Department of Health, Victorian Rural Generalist Training Program, Melbourne, VIC Australia

**Keywords:** Rural generalist training, Blended supervision, Remote supervision, General practice, Clinical supervision

## Abstract

**Background:**

Expanding rural training is a priority for growing the rural medical workforce, but this relies on building supervision capacity in small towns where workforce shortages are common. This study explored factors which support the use of blended supervision models (consisting of on- and offsite components) for postgraduate rural generalist medical training (broad scope of work) in small rural communities.

**Methods:**

Data were collected between June and August 2021 through semi-structured, in-depth interviews with medical training stakeholders experienced in blended supervision models for rural generalist training. Interviews were audio-recorded, transcribed verbatim and analysed using an inductive thematic analysis process.

**Results:**

Fifteen participant interviews provided almost 13 h of audio-recorded data. Four themes were developed: governance, setting, the right supervisor and the right supervisee. Blended supervision models may be effective if selectively applied including where the model is well-planned, the setting has local team supports and supervisor and supervisee characteristics are appropriate.

**Conclusions:**

Understanding factors involved in the application of blended supervision models can help with expanding rural generalist training places in distributed communities. Blended supervision models can be effective for rural generalist training if the model is planned, and the context is suitable.

**Supplementary Information:**

The online version contains supplementary material available at 10.1186/s12909-022-03529-x.

## Background

Globally, recruiting and retaining the health workforce in rural communities continues to be a challenge and rural training is known to be a major strategic pillar [1]. Although rural training places may seek to expand, as has been done in the general practice (GP) training program in Australia [2], the capacity to deliver this training in more distributed and smaller rural communities depends on having enough accredited supervisors to provide clinical supervision of registrars (trainee GPs) [3]. This is difficult to achieve in small rural communities that have an increased burden of recruitment and retention issues that put a limit on the number of locally qualified staff (fellowed GPs) who are eligible to support a rural generalist scope of teaching and learning [4]. An alternative to traditional models of face-to-face supervision is called blended supervision involving some face to face (on-site) and some remote (off-site) supervision of registrars. The principles (Table 1) of blended supervision models have been described as a way to expand rural GP training [2, 5]. However, there is limited empirical research about the factors related to the application of blended supervision models for postgraduate rural generalist medical training (where doctors cover primary care, emergency services and other advanced areas of practice) in small rural communities particularly from the perspective of the wide range of health service and training stakeholders who are involved.

**Table 1:** Principles of blended supervision models applied more broadly to GP training [2, 5]

Principles of blended supervision models applied more broadly to GP training [2, 5]
1. There is a blend of local and remote (offsite, technology-based) supervision
2. Local supervision (i.e. on-site) resources (e.g. onsite GPs, practice managers, Aboriginal health workers, practice nurses and allied health professionals), that provide the supervisee with face-to-face support and insights into the local context, are first identified and used
3. Off-site supervision and teaching are added to the local resources to ensure that the combined arrangement meets both patient safety requirements and registrar learning
4. All practices are unique, so blended supervision models will differ from one practice to another

Blended supervision is allowable under college standards for GP training in Australia [6, 7]. It is known to provide the opportunity for registrars (trainee GPs) to be placed in rural practices where learning opportunities are potentially diverse [8] when the practice is unable to meet all the traditional supervision requirements [2]. Blended models of supervising registrars need to be accredited on an individual basis and closely monitored by an external agency [6, 7]. The on-site component is the face-to-face support the supervisee (i.e. registrar or trainee) receives from the local doctors and other staff. The off-site component is typically provided by an accredited principal supervisor, who is usually located at another site and co-supervises with the on-site clinical team. The literature describes models where the off-site supervisor spends some time initially with the supervisee face-to-face to build the supervisory relationship and to understand the supervisee’s workplace context [2]. Subsequently, the supervisor provides support from a distance using videoconferencing or telephone. However, there is limited empirical data about the factors which support the use of blended supervision models for the wide range of stakeholders involved in rural generalist medical training.

### Research context

In Australia GPs, like all doctors in training, require clinical supervision and assessment to complete qualifications. Australia is interested in the blended supervision phenomenon as it has introduced a National Rural Generalist Training Program [9]. Rural generalists are doctors who have developed skills that enable them to safely deliver a breadth of comprehensive primary care, emergency and other specialist services in rural and remote communities that have access to fewer individual specialists [9]. These doctors are more likely to work in communities of 15,000 or less compared with GPs who only work in general practice [10]. However, Australia faces a major shortage of such doctors, posing an urgent need to explore expansion of supervision of postgraduate doctors on rural generalist career pathways. Their scope of work covers general practice as well as the typical procedural (obstetrics, anaesthetics, emergency, surgery) and non-procedural (general medicine, Aboriginal and Torres Strait Islander health and palliative care) caseload which is required in the distributed communities where rural generalists will eventually work [11, 12]. Rural generalist GPs are the main doctors who supervise rural generalist trainees, however, other specialists in procedural and non-procedural fields contribute to teaching and learning and signing off trainee competencies. Supervising at a generalist scope of work in small community contexts may present nuanced challenges for the blended supervision model as the work of rural generalists is far broader than traditional general practice. Therefore, the aim of this study was to explore the factors which support the use of blended supervision models for postgraduate rural generalist medical training in small rural communities.

## Methods

### Definition of terms

Firstly, the research team agreed on the project terminology (Additional file 1 ) to support standardised interpretation.

### Research design

This study, based in the Australian state of Victoria, was designed using a constructivist lens, where participants and the interviewers co-constructed meaning [13]. Individual, semi-structured interviews were used to elicit richer, in-depth information about stakeholders experiences with blended supervision models in rural generalist work settings. Interviews were chosen over focus groups to allow for scheduling and practical considerations given the stakeholders were based in different states and in multiple time zones.

### Procedure and data collection

The peak agency involved in supporting supervision for GP training, GP Supervisors Australia (GPSA), identified relevant stakeholders from their extensive contacts across government, industry, and academia, including those involved in the independent program, Remote Vocational Training Scheme (RVTS), remote and rural training organisations and health services, supervisors and academics with expert practical knowledge of blended supervision models for generalist scope of work in rural settings. These stakeholders were known to GPSA from their ten-year history of supporting GP training. Potential participants (*n* = 44) were invited by email and phone (SMS). Participants read the explanatory statement online and submitted written consent whereby they selected an interview time with one of three trained qualitative interviewers, two of whom had a non-GP clinical background and academic expertise in supervision (PM and BOS) and one who had a business and systems-focused background (CT). None had prior relationships with the participants but two (PM & BOS) had a background in research about supervision models. Three email reminders were sent by GPSA to follow-up.

The interview guide (Table 2) was developed in line with the existing clinical supervision and GP training literature and was piloted with a project advisory group, inclusive of regional coordinators of GP training and health service executives from three Victorian rural generalist training regions. Questions in the interview guide were suitable to both health services and GP supervisors and those that were in management or other roes related to GP training and supervision. This latter group was encouraged to draw upon their experience with developing, implementing, evaluating, accrediting or supporting blended supervision models. The interview guide was circulated to participants prior to the interview to support deeper reflection. This approach has been used successfully in previous interview studies conducted by the first author. All interviews were conducted via zoom or telephone and audio recorded then transcribed. The duration of interviews was determined by participants and ranged between 35 and 74 min. Except for one interview with a pair, the remaining were individual interviews. There was no payment for participating.

**Table 2:** Interview guide

What is your experience with blended supervision models and where the trainees and supervisors may work across multiple sites and supervision is not necessarily occurring face to face? Where, when were you involved in these models?
Have you applied these models training RG doctors (scope of general practice, admitting to hospital, seeing inpatients, and doing emergency on-call)?
If so, what was the scope of supervision you are aware that could be supported remotely [prompt: caseload per week, frequency/duration spent on rosters]
What part was supported face to face? [of the above scope]
What level of backup was used if the doctor needed help?
What educational supports were used?
How was technology used, for example, phone or video, or document sharing, to connect with the trainee?
More specifically, how did you manage restorative supervision to help with coping strategies, stress management, burnout, debriefing?
How did you manage supervision for formative skills and knowledge development and learning guidelines, ethics, and norms?
How did you know that the patient was safe? [prompt: in terms of learner safety, the supervisor may have the role of orientating learners, being available to respond to a registrar’s clinical questions during consulting hours, conducting audits of registrar patient care, such as random case analysis, responding to critical incidents and complaints.]
How did you know that the learner was learning? [prompt: In terms of learning, supervisors would also be responsible for developing and reviewing the learning plan, facilitating educational opportunities that evolve from clinical work, and providing tutorials.]
Did you have any near misses whilst using blended supervision models? If so, what happened and what did you learn from these?
How were the models evaluated and what were the outcomes [satisfaction by learners, supervisors, impact on patient care]?
How easy were these models to accredit – do you have any tips there?
In summary, what are your three top tips for enhancing the effectiveness of blended supervision models for those that are new to this?
Is there anything else you would like to add?

### Data analysis

Each interviewee was assigned a unique identifier. The research team sought to interpret emergent findings using inductive thematic analysis. Firstly, the research team read the full transcripts, re-reviewed recordings, and independently coded the data for meaning [14]. This happened with no pre-set coding frame, in line with inductive analyses processes. Additions and alterations to the codes were made as blocks of five transcripts were completed. Authors then double-coded another transcript identifying reasonable concurrence with the codes and adding extra codes if these were relevant. The material was discussed, annotated, and then organised into emerging themes, layering, and reorganising these to make sense of the data [15]. All stages of analyses occurred with the research team working in distributed sites and meeting weekly online, to challenge each other’s ideas, reduce subjective biases and test any assumptions [16].

### Ethics approval

This research had ethical approval from the Human Research Ethics Committee of Monash University (project no: 29263).

## Results

Fifteen participants were interviewed who were either GPs with a role in supervision provision at rural generalist scope or those not trained as doctors and working in roles related to supporting, or accrediting GP training at rural generalist scope. Most participants held more than a single role and were asked to reflect on the role/s closest to blended supervision models for the purpose of the interview. Nearly 23 h of audio-recoded data were yielded from the interviews.. More information about the demographic characteristics of participants is included in Table 3. Thematic analysis of data identified four themes namely, governance, setting, the right supervisor and the right supervisee. Themes and sub-themes have been presented in Table 4.

**Table 3:** Demographic characteristics of participants

Variable	*N* (total = 15) 32% response rate
Gender	
Male	5
Female	10
Role	
GP	7
Management/other	8
Location	
Victoria	9
Other states	6

**Table 4:** Themes and sub-themes

Theme	Sub-theme
Governance	Agreed roles and responsibilities
	Clear communication systems, including escalation methods fit to the setting
	Quality improvement processes
Setting and scope of services	Team supports
	Community of practice and social supports
	Scope of services amenable to blended supervision
The right supervisor	Characteristics of the right supervisor
	Medico-legal risks
	Reward
The right supervisee	Characteristics of the right supervisee
	Invested in the training location

### Governance

Participants, both GPs and those in education and management roles, felt that based on their real-world experience, blended supervision models in distributed communities were enabled by have clear governance systems in place. This involved having agreed roles and responsibilities of all parties involved, establishing clear communication systems including escalation methods and having quality improvement processes around the blended supervision model in use.

#### Agreed roles and responsibilities

A major factor enabling blended supervision models was having agreed roles and responsibilities around the rural generalist scope of work. Participants emphasised the need for clarity of roles, of both the on-site and off-site supervisors, and the importance of having others on-site that can offer support where required. One participant said:

*“…need to have absolute clarity around what the off-site supervisor is actually going to do including a position description for the off-site supervisor…when I think back to the other model the registrar had an off-site supervisor…there was always somebody (in the clinical area), even though they're not their supervisor…[so] there is someone that they can ask clinical advice of regardless.”* **Int 8.**

#### Clear communication systems including escalation methods fit to the rural generalist training setting

Participants felt that it was imperative that supervisor-supervisee communication systems were clear, and tailored to the formative stage of rural generalist learners and accounted for both incidental and formal learning needs:

*“At the beginning, there needs to be some discussion about every single patient… it’s set up so that the supervisor knows, has a really good opportunity to see what the scope is and that some of that is direct observation.”* **Int 13**

Nothing could be taken for granted, and agreement was needed around escalation processes specific to the isolated context that rural generalists work in.

“*What does that plan of supervision look like? how often, when, how do you know if it's an emergency? Who is your contact? That needs to be put in place…it needs a Plan B and a Plan C”* **Int 5**

It was also deemed important to identify at the outset the capabilities of the local setting, and ensure the trainee understood the boundaries around when and how to act when something happened in the context of having limited medical staff in small towns.

*“… in some of the small towns, it's having a plan for getting out of trouble. So, who do you call? Who do you normally refer to? Where's your retrieval site? It's looking at those types of things. Partly it’s being aware of what facilities there are in the location… nursing staff backup …how do you get onto your retrieval people? And where do you send people?”* **Int 3**

#### Quality improvement processes

Quality improvement systems were considered essential for blended supervision models given the fragility of rural healthcare, related to staff recruiting and turnover, may require changes to the model over time:

*“Because what you're really doing is you're trying to set up a system for when things go wrong… Who is the registrar going to go to if things go wrong? Who is the supervisor going to go to if things are going wrong?”* **Int 13**

Having an external agency managing the remote supervision model made it easier to respond to near misses and remove a rural generalist trainee if warranted:

*“…reflecting on what works, what doesn't… it's constantly reviewed. And at any point if it is questionable… there's an opportunity to remove or withdraw. Yeah, sometimes you just don't know until you're in there”.* **Int 5**

### Setting and scope of services

Participants with a medical background (i.e. GPs), including those from public health settings, and those in roles supporting GP training described settings most suited to blended supervision models as those characterised by having team supports, encouraging communities of practice and social supports, and having a defined scope of work that was amenable to blended supervision.

#### Team supports

The training post was organised as a whole multidisciplinary team learning model with a variety of learning and feedback opportunities for the generalist scope of work:

*“It’s important to have feedback from people around the trainee, so … the supervisor is having that close connection maybe with other doctors that are working in the environment, or … the nurses who are working with them, and having some sort of multisource feedback”* **Int 14**

Multi-learner practices were also valued for being:

*“part of the teaching and learning process … they can pass on knowledge, and they can pass on an understanding”.* **Int 12.**

#### Communities of practice and social supports

For stakeholders more familiar with remote supervision for trainee generalists working in isolated locations, setting up a community of practice and social supports for the learner was a fundamental part of a blended supervision model:

*“It's not just about what happens in the practice, it's about, you know, this doctor is probably going to be living in this community. So, what social supports are there, how are they going to be integrated into the community?” ***Int 9**

#### Scope of services amenable to blended supervision

Non-procedural services were considered more suited to a blended supervision model, whereas the procedural services of rural generalists posed risks:

*“Well I mean [x] is two and a half hours from the nearest hospitals, so…if someone had a life-threatening illness, you'd never get any one to them, we had to manage the first two and a half or three hours”.* **Int 1**

Blended supervision also needed to be set up to cope with the undifferentiated workload of rural generalist care:

*“you might last night have been the junior doctor, helping the senior with an MVA [motor vehicle accident]. But tomorrow, you're doing coughs and colds”.* **Int 13.**

### The right supervisor

Participants that were GPs working in health administration, training and education roles described the characteristics of a right supervisor, suitable to providing blended supervision. The right supervisor was noted to be someone with sound knowledge of the supervisee’s work context and community, awareness of the trainee’s skillset in relation to the requirements of the specific rural or remote context and ability to foster a positive supervisory relationship with the supervisee. There were also considerations of medico-legal risks and the importance of being rewarded.

#### Characteristics of the right supervisor

The right supervisor for blended supervision was seen to be one with good knowledge of the trainee’s community:

*“I think knowing this setting, I don't think I could have done that if I'd never lived in [x]… I'm not going to suggest you get a CT scan, because I know that there isn't a CT scanner in town.”* **Int 13.**

Further they were supervisors who could build a positive relationship with the learner:

*“meet[ing] up with them and try and meet up with their family … ideally in their own town…or face to face on a Zoom so that we've got that connection…and share my background so there’s that comfort, shared experience”.* **Int 2.**

And who understood the trainee’s skills relative to the requirements of the specifics of the learning context:

*“Early on in those situations the supervisor needs to have that understanding…Yeah, context and service and setting”* **Int 3**

#### Medico-legal risks

Supervisors involved in the blended supervision models (off-site component of rural generalist supervision) required a tolerance of medico-legal risks to the supervisor, including seeing these as similar to those of on-site supervisors:

*“…I think that it’s not specific to off-site supervisors. It’s something that happens with on-site supervisors as well, where you might pick things up that aren’t safe only when you’re actually sitting with a trainee or observing them in practice.”* **Int 14**

#### Reward

Remote supervisors saw wider value than financial reward for supervising but they equally like having some funding to recognise their effort:

*“You have to ask what's in it for the supervisor, I mean there has to be something, it doesn't necessarily need to be money in it, but if it's not money, it's got to be some other thing that makes it worthwhile…”* **Int 1.**

### The right supervisee

Participants, both GPs and non-GPs working in GP training and education roles, described the characteristics of a ‘right’ supervisee (i.e. trainee/registrar), suitable to receiving blended supervision for rural generalist scope of work. This was viewed as someone that was interested and invested in the rural generalist work context, who had good insight into their skillset to identify areas requiring help or feedbcak, having the ability to seek out help as needed, and possessed good levels of confidence and resilience. Trainee’s commitment to the training location was also considered important.

#### Characteristics of the right supervisee

The formative stage of the rural generalist trainee and their skills and knowledge were essential to understand in the context of working in more isolated settings. Trainees deemed suitable were those with a baseline:

“*skillset or a level of skill, where they know when they can deal with something and when they can't…it's actually making sure that we have the right people in the right place.”* **Int 8.**

The registrar’s knowledge of the community and context was considered just as important as having technical medical skills:

*“If you have been in [this region] before, so if you've done your medical training [here], or you grew up [here], or you may have been in a very similar context before, they may consider you [as an early-stage registrar]. But if you're coming fresh out of Sydney [city], or Adelaide [city], wanting to do a GPT1 (first stage of community GP training) remote, the chances are the training post won't take you on”*. **Int 4**

The trainee’s learning style, confidence levels and their fit to the blended supervision model within the rural generalist context of work was important:

*“Level of confidence really needs to be right. Sometimes they can be overconfident… if somebody is overconfident that can be risky and if somebody is under confident that might also impact their judgement…”* **Int 5**

The learner’s resilience could also determine the success, or otherwise, of a blended supervision model:

*“…some who, if you put them in a remote supervision model, are going to be so anxious and feel really unsupported…. they may actually require more support early on to make sure they don't have an awful experience and want to give it all up.”* **Int 14**

#### Invested in the training location

Blended/ remote supervision was enabled if trainees were invested in training in the rural or remote location. One supervisor noted:

*“they all wanted to be there, and we were understaffed… The ones who bothered applying for three months in [x] tended to be pretty enthusiastic and would last the three months easily.”* **Int 1.**

## Discussion

Blended supervision models are much needed in the rural medical training pipeline, to ensure access to rural generalist training in distributed communities. This study explored the factors related to the use of blended supervision models, drawing on the perspectives of a wide range of stakeholders involved. This is important where there are so many stakeholders who are involved in rural generalist training each of whom needs to buy in to proposed training models. The paper specifically informs the context of supervising a wider scope of (procedural and non-procedural) work in the context of learning in small rural towns. By doing so, it provides timely information for setting up successful blended models of supervision across a rural generalist scope to expand training places in distributed communities. Findings indicate that blended supervision models can be set up to work successfully, however, a range of planning needs to be done before realising these models. This involves documenting governance, establishing supportive work environments and identifying the right supervisor and supervisee as pre-requisites for successful blended supervision models for rural generalists. Whilst these principles also apply to ensuring effective face-to-face clinical supervision [17], our study findings highlight they are more prominent in a blended supervision model, in order to work effectively where the supervisor is not on-site to make immediate adjustments and the trainee may be working in relatively isolated situations with an undifferentiated caseload [18].

Whilst there are some frameworks on providing remote clinical supervision in postgraduate medical training [5, 18], and on undertaking clinical supervision using technology [termed ‘telesupervision’; 19], empirical data about the real-world implementation of any form of off-site supervision models for rural generalist training environments has been scarce. Building on previous literature about the principles of blended supervision, this study builds evidence about how blended supervision models work in actual practice and a rural generalist context. It is unsurprising that participants reiterated the importance of having sound governance, given the inherent medico-legal risks that an off-site or remote supervisor may face, as well as the need to be proactive around achieving learning goals. Strong governance needs to be underpinned by the careful selection of supervisor/s and the supervisee, as it is likely that not every rural generalist trainee is suited to blended supervision [19, 20]. The findings in this study support the attributes of an effective supervisor found in the literature [17, 21] showing that an effective supervisor in a blended model needs experience, be available, good at building a supervisee’s trust and relationship, and understand the supervisee’s work context and emerging skill levels. They also need to be able to handle some level of uncertainty [17, 18, 21].

This study also highlights the need for the supervisee within a blended model of supervision to have certain attributes: possess sound knowledge of the community, willingness to learn and grow, not hesitant to ask questions, be resilient, cope with change and uncertainty, take responsibility for own learning and recognise limitations. This is important in rural settings where trainees can encounter a wide caseload and might gain from rich opportunistic learning [21]. These characteristics are highly important as previous research has revealed the risk of supervisees’ missing out on formative development when undertaking telesupervision, especially if they do not assume responsibility for their learning, which could ultimately have an adverse impact on patient care [20].

The work setting where the supervisee undertakes blended supervision is also an important consideration. This study suggests that blended supervision models work well for rural generalist scope, when there are good supports from others in the team, including other medical practitioners, nurses, or allied health professionals. Such team members can be good sources of information about the local context and can provide day-to-day support, especially when the off-site supervisor is unavailable (e.g., for an urgent question that has emerged or debriefing). A well-known example of this is seen in the Rural Vocational Training Scheme (RVTS), where doctors are supported on-site by nurses whilst outreaching into remote and isolated Aboriginal and Torres Strait Islander communities in Australia [22]. Given the rising importance of interprofessional collaboration in managing complex health care needs [23], utilising team support structures in blended supervision models is time well-invested.

The researchers employed a number of measures to enhance the study’s trustworthiness. This included the use of reflexivity, piloting the interview guide, and co-analysis and triangulation of data between all the researchers. The first and second authors (PM and BOS) are well-renowned clinical supervision researchers, and it is likely that some participants were aware of them. It is likely that the second, third and fourth authors (CT and GW) in their respective roles were known to some GP supervisors. To mitigate the extent of these potential influences on participant’s perspectives and sharing of responses, all the interviews followed the pre-established interview guide. The researchers met regularly during the course of data collection and analyses to have transparent discussions about their ongoing experiences with the study to ensure transparency and reflexivity [15].

### Strengths and limitations

This study has some strengths and limitations. It is the first in-depth study to explore the factors related to the implementation of blended supervision models, across a wide scope of rural GP training in small communities, from multi-stakeholder perspectives. Whilst the principles governing blended supervision models were explored, the types of technology used and decision-making around the use of blended models, wasn’t explored in-depth.

### Implications for practice and research

Information from this study can be used by the National Rural Generalist Training Program, levels of government, GP Colleges, health services and supervisors to implement best practice blended supervision for rural generalist training expansion. This includes scanning local supervision capacity, screening eligible trainees, building technology and planning resources and developing quality management systems. Participants in this study were sampled from the medical education community, thus creating a need for testing the applicability of these findings in other professions where blended models of supervision are used, such as rural allied health providers who work at generalist scope [24]. Sustainability of blended supervision models still remains to be researched.

## Conclusion

This study explored factors which support the use of blended supervision models, across a wide scope of rural generalist training in small rural communities, from a range of stakeholder perspectives. Blended supervision models, that typically include an off-site (remote or telesupervision) component, can be set up to work well if some pre-requisites are met. Establishing good governance around the model, choosing the right setting, supervisor, and supervisee to engage in the model is of paramount importance. Further research is needed to understand the enablers of and barriers to sustainability of blended supervision models in rural generalist training and how these models work in professions other than medicine.

## Supplementary Information

**Additional file 1:** Definition of terms applied to the project.

## Data Availability

Data is protected by ethics. All reasonable requests to access de-identified data can be made in writing to the corresponding author.

## Abbreviations

GP: General Practice
GPSA: General Practice Supervisors Australia
RVTS: Remote Vocational Training Scheme
