# Supplementary material for: Title-blended supervision models for post-graduate rural generalist medical training in Australia: an interview study
Source: BMC Med Educ. 2022 Jun 20;22:478. doi: 10.1186/s12909-022-03529-x (PMC9210640; doi:10.1186/s12909-022-03529-x)
Supplement: Supplementary file 1 — Additional file 1: Definition of terms applied to the project. [file 12909_2022_3529_MOESM1_ESM.docx]

**Appendix 1: Definition of terms applied to the project**

| **Term** | **Definition** |
| --- | --- |
| Clinical supervision | The regular structured extended encounters that happen one to one which involves intensive relationship-based education and training that is work-focused, and which manages, supports, develops, and evaluates the work of colleagues [25].  The supervision partnership addresses functions of quality control, maintaining and facilitating competency, and helping supervisees to work effectively [25].  It includes aspects of both mentoring and coaching – supporting someone’s personal and professional development (e.g., mentoring) as well as looking after their performance (e.g., remedial supports) [26].  It is also much wider than educational supervision which is about reviewing progress in a training program [26]. |
| Rural community | Rural communities are defined as Modified Monash Model 2-7 [27]. In smaller rural populations, from MM4+, or 15,000 population or less, a higher proportion of GPs works in procedural areas of care (emergency, surgery, anaesthetics and obstetrics and gynaecology) [10]. |
